# State-to-State Quantum Dynamics Study of Intramolecular Isotope Effects on Be(^1^S) + HD (*v*_0_ = 2, *j*_0_ = 0) → BeH/BeD + H/D Reaction

**DOI:** 10.3390/molecules29061263

**Published:** 2024-03-13

**Authors:** Hongtai Xu, Zijiang Yang

**Affiliations:** School of Physics and Electronic Technology, Liaoning Normal University, Dalian 116029, China

**Keywords:** quantum dynamics, time-dependent wave packet, intramolecular isotope effect, integral cross section

## Abstract

The dynamic mechanisms and intramolecular isotope effects of the Be(^1^S) + HD (*v*_0_ = 2, *j*_0_ = 0) → BeH/BeD + H/D reaction are studied at the state-to-state level using the time-dependent wave packet method on a high-quality potential energy surface. This reaction can proceed along the indirect pathway that features a barrier and a deep well or the smooth direct pathway. The reaction probabilities, total and state-resolved integral cross sections, and differential cross sections are analyzed in detail. The calculated dynamics results show that both of the products are mainly formed by the dissociation of a collinear HBeD intermediate when the collision energy is slightly larger than the threshold. As the collision energy increases, the BeH + D channel is dominated by the direct abstraction process, whereas the BeD + H channel mainly follows the complex-forming mechanism.

## 1. Introduction

Isotope substitution provides important insights into the dynamic mechanisms of chemical reactions. In particular, the intramolecular isotope effects that are generally represented by the resulting product branching ratio of an elementary reaction can provide more significant details about reaction dynamics and are often used to investigate bond-selective processes and identify reaction pathways [1,2,3,4,5,6]. Triatomic A + HD reactive systems, as the simplest example, have been extensively investigated in both experiments and in theory [7,8,9,10,11,12,13,14,15,16]. It was argued on the basis of these studies that the AH/AD ratio for a complex-forming reaction that has a well on the reaction pathway is less than one, whereas the direct abstraction reactions dominated by an activation barrier usually feature an AH/AD ratio larger than one. This bias can be attributed to the mass asymmetry of the HD molecule, resulting in a biased cone of acceptance and the molecular reorientation effect.

The intramolecular isotope effects or the branching AH/AD ratios are extremely affected by the topography of the corresponding reactive potential energy surface (PES). In 1999, Skouteris et al. studied the role of the van der Waals well in the entrance channel of the Cl + HD reaction by exact quantum dynamic calculations on two different PESs and crossed molecular beam devices [17]. The results showed that the shallow well (~0.02 eV) can cause a strong preference for the DCl product, and the production of the DCl/HCl ratio is enhanced more than seven times for low rotational excitation HD when the very weak van der Waals interactions are included, which is also in agreement with the experimental measurements. Wu et al. studied the significance of conical intersections in the C(^1^D) + HD reaction by using a continuous supersonic flow reactor combined with the quantum dynamics and quasi-classical trajectory calculations on an adiabatic ground-state PES [18]. A barrier on the reaction path induced by the conical intersection between the $\tilde{b}$^1^*A*″ and $\tilde{d}$^1^*A*″ states can regulate a special metastable-state intermediate before the long-lived complex is formed in the deep well, resulting in the CD/CH product branching ratio markedly increasing. Two unusual mechanisms with obvious nonstatistical features, namely C-H activation complex conversion and cyclic complex, were found by the trajectory analysis. In addition to the adiabatic ground-state reactive process, strong intramolecular isotope effects also exist in excited state reactions. Recently, quantum dynamics research on the nonadiabatic reaction of electronically excited Be^+^(^2^P) with HD molecule revealed that the shallow wells induced by the avoided crossing on the diagonal surface bring a dramatic preference for the BeD^+^ product [19]. It can be concluded from the calculated results that the formation of BeH^+^ favors a direct reaction process, whereas the BeD^+^ + H channel is dominated by the complex-forming mechanism. Similar dynamic behaviors were also presented in the nonadiabatic Mg^+^(^2^P) + HD reaction [20], which are consistent with the previous experimental results [21].

For the A + HD type reaction, there usually exists a dominant well or barrier that determines the preference for the production of AH or AD on the reaction path. However, the reactions of ground-state alkaline earth metal with hydrides (Be + H_2_, Mg + H_2_, and Ca + H_2_) proceed either directly through hydrogen abstraction or indirectly via an intermediate complex [22,23,24,25,26]. The complex-forming reaction paths include both an obvious well and a barrier structure, and the molecular systems were found to be linear at equilibrium, different from the T-shaped stable configuration of typical complex-forming reaction systems of CH_2_, OH_2_, and SH_2_ [27]. Therefore, the reactions between alkaline earth metal atoms and HD molecules may present unconventional product branching ratios and interesting dynamic mechanisms, which have not been studied until now. As the simplest example of this type of system, the BeH_2_ has received an important amount of attention because of its simple electronic structures and has been considered a good testing tool for new computational methods of quantum chemistry. The structure factors, vibrational mode, and infrared emission spectroscopy of the BeH_2_ molecule, and the BeD_2_ and BeHD isotopomers, have been widely studied by various experimental technologies and ab initio calculations [28,29,30,31,32]. However, the reaction dynamics of the Be atom with H_2_ was not implemented until recently. In 2022, Yang and Chen reported a globally accurate ground-state BeH_2_ PES based on a mass of ab initio points calculated at the icMRCI + Q/AV5Z level and the neural network method [33]. On this new PES, the quantum dynamics of the Be(^1^S) + H_2_ (*v*_0_ = 0, *j*_0_ = 0) → BeH + H reaction were carried out, and the dynamics results indicated that the reaction follows the complex-forming mechanism near the reactive threshold, while a direct abstraction process gradually plays the dominant role as there is an increase in collision energy.

In this work, we perform state-to-state quantum dynamic calculations of the vibrationally excited Be(^1^S) + HD (*v*_0_ = 2, *j*_0_ = 0) → BeH/BeD + H/D reaction using the time-dependent wave packet (TDWP) method on the newly constructed PES to study the dynamic mechanisms. To initiate the reaction at the lower collision energy and analyze the microscopic dynamic behaviors and intermolecular isotopic effects in a wider energy region, the initial vibrational state of the HD molecule is set to two due to the large endothermicity (~2.45 eV) of the title reaction with the vibrational ground-state. The remainder of this paper is organized as follows: Section 2 introduces the theory and computational details; the calculated results are listed and the corresponding discussions based on these results are given in Section 3; and the conclusions of this work are displayed in Section 4.

## 2. Results and Discussion

**Figure 1:**
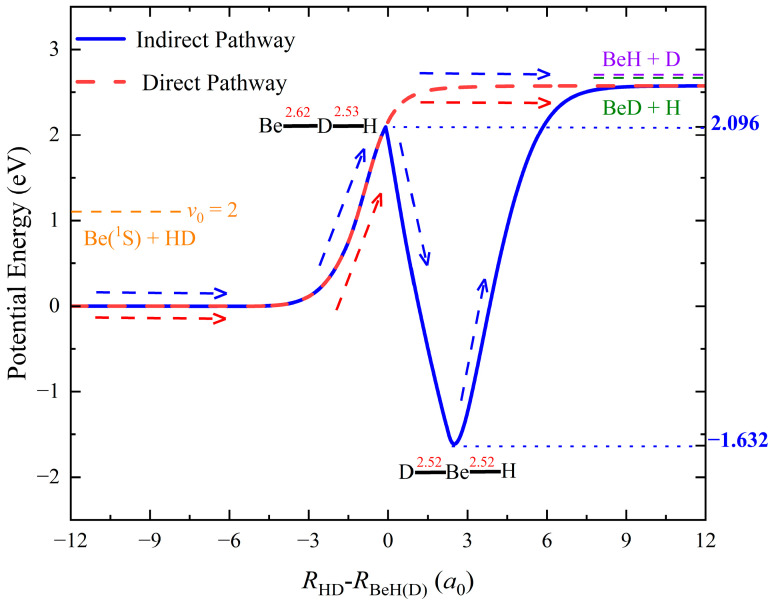
Indirect and direct pathways of the Be(^1^S) + HD (*v*_0_ = 2, *j*_0_ = 0) → BeH/BeD + H/D reaction.

The TDWP dynamic calculations of the Be(^1^S) + HD (*v*_0_ = 2, *j*_0_ = 0) → BeH/BeD + H/D reaction are carried out on the ground-state PES [33] constructed by the permutation invariant polynomial neural network model [34,35] based on high-level ab initio calculations. The used PES includes all the regions that the reaction can access and features high precision; thus, it can be used to accurately calculate the state-to-state dynamics of the Be(^1^S) + H_2_ reaction and its isotopic variants. Figure 1 presents the indirect and direct microscopic pathways of the Be(^1^S) + HD reaction, which are obtained by scanning the ground-state PES with small step lengths (Δ*R* = 0.01 *a*_0_, Δ∠Be-HD = 1°) at different coordinates (*R*_HD_–*R*_BeH(D)_) to find the minimum energy. The indirect pathway is also the global minimum energy path, which includes a barrier and a deep well with a depth of 1.632 eV corresponding to the reactant channel. The Be atom collides with the HD molecule in the collinear direction. It passes a tiny transition state that is 2.096 eV above the energy of the reactant asymptotical region. Then, the Be atom moves along the mid perpendicular of the HD molecule together with the elongation of the H-D bond. When the system is at *D*_∞*h*_ symmetry, a stable H-Be-D complex is formed; finally, the product BeH/BeD molecule is generated via the dissociation of the complex at the product asymptotical region. The H atom more easily escapes the constraint of wells due to the lighter mass, so the formation of the BeD product is dominant. There is no well or barrier on the direct reaction pathway, and the energy is monotonically increasing from the reactant channel to the product channel. When the reaction process is on the direct abstraction pathway, the H atom describes a wider circle around the center of the mass of the HD molecule and is more likely to intercept the approaching Be atom. Considering the zero-point energy effect, the endothermicities of forming BeH + D and BeD + H are 1.59 eV and 1.56 eV, respectively.

**Figure 2:**
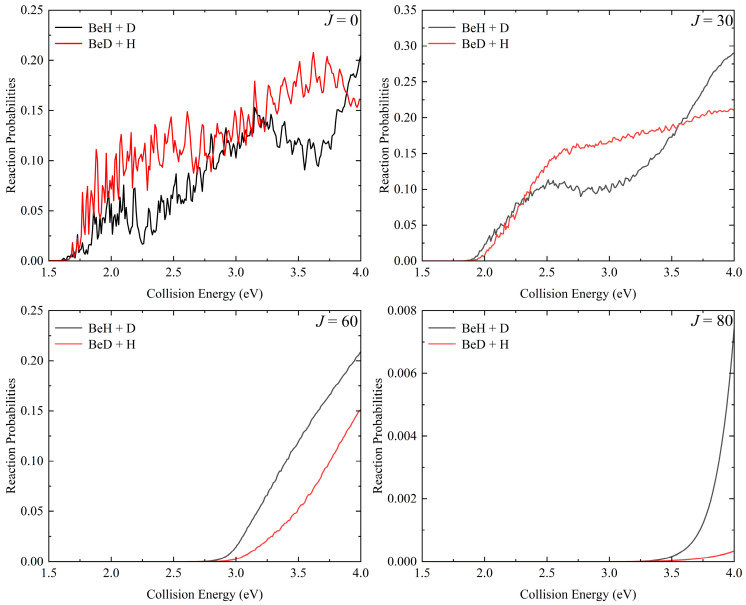
Total reaction probabilities of the Be(^1^S) + HD (*v*_0_ = 2, *j*_0_ = 0) → BeH/BeD + H/D reaction as a function of collision energy at four particle waves (*J* = 0, 30, 60, and 80).

The total reaction probabilities as a function of collision energy for the Be(^1^S) + HD (*v*_0_ = 2, *j*_0_ = 0) → BeH/BeD + H/D at four different partial waves (*J* = 0, 30, 60, and 80) are displayed in Figure 2. For *J* = 0, the reaction threshold of the two channels is consistent with the corresponding endothermicity determined by the PES. There are prominent oscillations on the reaction probability curves, especially near the threshold, which are attributed to the formation of a long-lived HBeD complex in the deep well of the indirect pathway that can support numerous bound and quasi-bound states. The threshold increases and the oscillation peaks become wider as there is an increase in the *J* value because the increasing centrifugal barrier decreases the depth of the effective potential well. It can be seen that the reaction probabilities of the two channels present different behavior at a certain *J* value. For the BeD + H channel, the reaction probabilities decrease with the increasing *J*, but the probabilities have larger values at high partial waves for forming the BeH product when the collision energy exceeds the reaction threshold of the corresponding partial wave even though the effects of the centrifugal barrier increase. In addition, the reaction probabilities of the BeH + D channel are obviously larger than the BeD + H channel at high *J* values, which is because the higher zero-point energy of the BeH product makes it easier to overcome the centrifugal potential. The well is gradually smoothed by the centrifugal potential, resulting in the reaction processes via a direct abstraction pathway.

**Figure 3:**
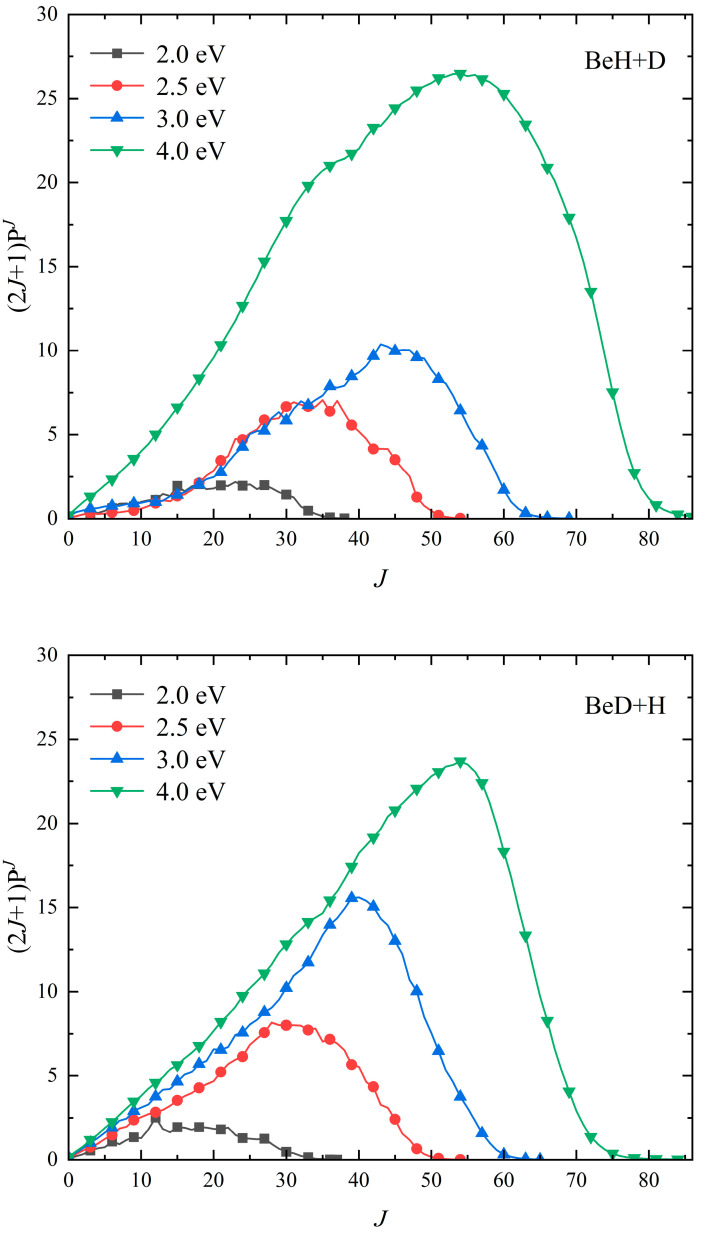
Opacity functions of the Be(^1^S) + HD (*v*_0_ = 2, *j*_0_ = 0) → BeH/BeD + H/D reaction at the collision energy of 2.0, 2.5, 3.0, and 4.0 eV.

To show the contribution of each partial wave on the cross section of the Be(^1^S) + HD (*v*_0_ = 2, *j*_0_ = 0) reaction, the weighted opacity functions multiplied by (2*J* + 1) on the reaction probability at four collision energies (2.0, 2.5, 3.0, and 4.0 eV) for both of the product channels are shown in Figure 3. The BeD + H channel has a faster convergence than the BeH + D channel at a certain collision energy because the centrifugal barrier is lower when the Be atom attack occurs on the H side of the HD molecule at a given *J* value. All the probability distributions are dominated by a single maximum and barely present the oscillating structure, such as the C + HD reaction [18], as it corresponds to the fact that the well on the reaction path is not very deep, and the direct pathway also plays a crucial role, especially at relatively larger collision energies. For the BeD + H channel, the curves rise almost linearly with the *J* value and decrease abruptly near the maximum available partial wave, which is consistent with the complex-forming mechanism. The magnitude of the BeH + D channel shows a flatter increase and a more gradual drop after reaching the peak value, implying the nonstatistical dynamics behavior for the product BeH molecule.

**Figure 4:**
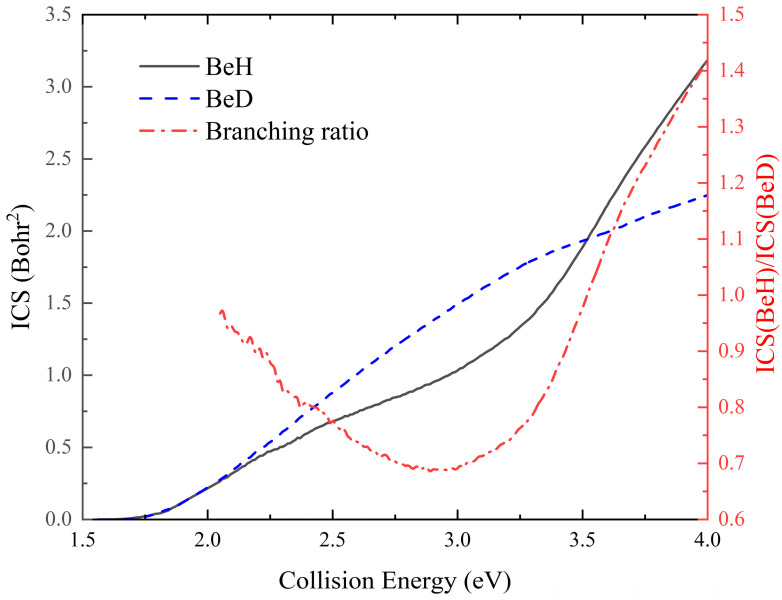
Total ICSs of the Be(^1^S) + HD (*v*_0_ = 2, *j*_0_ = 0) → BeH/BeD + H/D reaction and the BeH/BeD branching ratio as a function of collision energy.

The collision energy dependence of the total ICSs of the two product channels and the product BeH/BeD branching ratio for the Be(^1^S) + HD (*v*_0_ = 2, *j*_0_ = 0) reaction are shown in Figure 4. The left and right ordinates represent the ICS value and the ICS (BeH/BeD) branching ratio, respectively. Compared to the reaction probability curves of a single *J* value, most of the oscillations are erased by summing over various partial waves. The total ICSs of the two channels increase in a monotone way with the collision energy, conforming to the characteristics of endothermic reactions. However, the two channels display different variation behaviors. The ICS curve of the BeH molecule increases almost linearly when the collision energy is below 2.90 eV, and its rising slope gradually becomes larger as the collision energy continues to increase. The ICS of the BeD + H channel is also nearly linear, increasing below 3.20 eV, and then the curve rises slowly. These results suggest that the dominance of the title reaction changes from forming the BeD product to the BeH molecule as the collision energy increases.

It can be seen that the branching ratio is around one when the collision energy is slightly larger than the reactive threshold. This is because the reaction at relatively low collision energy mainly proceeds along the global minimum energy path, and a long-lived linear HBeD complex can be formed in the deep well, resulting in the two product channels being equally distributed. As the collision energy increases, the contribution of high-order partial waves increases, as shown in Figure 3, and the depth of effective potential on the reaction pathway is decreased, so the lifetime of the complex becomes shorter. As previous studies on C(^1^D) + HD [18] and Be^+^(^2^P) + HD [19] reactions have shown, the shallow well can regulate the intermediate to effectively improve the AD/AH ratio. Because the H atom moves faster than the D atom, thus the Be-H bond can be elongated, leading to the complex having smaller vibrational amplitudes in the Be-D bond stretching, so the BeD molecule is formed more easily, and this effect can be enhanced for a shallower effective well. Therefore, the BeH/BeD branching ratio first decreases with the increase in collision energy. As the collision energy continues to increase, the proportion of the collision along the direct abstraction pathway increases, and the effective well on the indirect path can be smoothed by higher-order partial waves, thus the branching ratio starts to rise. In addition, the orbital angular momentum *l*-dependent centrifugal barrier in the BeH + D channel increases slower than the BeD + + H channel due to the larger reduced mass of the former, which can also increase the BeH/BeD ratio. The preference for forming the BeH molecule is larger than the BeD molecule when the collision energy is above 3.56 eV, suggesting that the direct abstraction process starts to dominate the title reaction.

**Figure 5:**
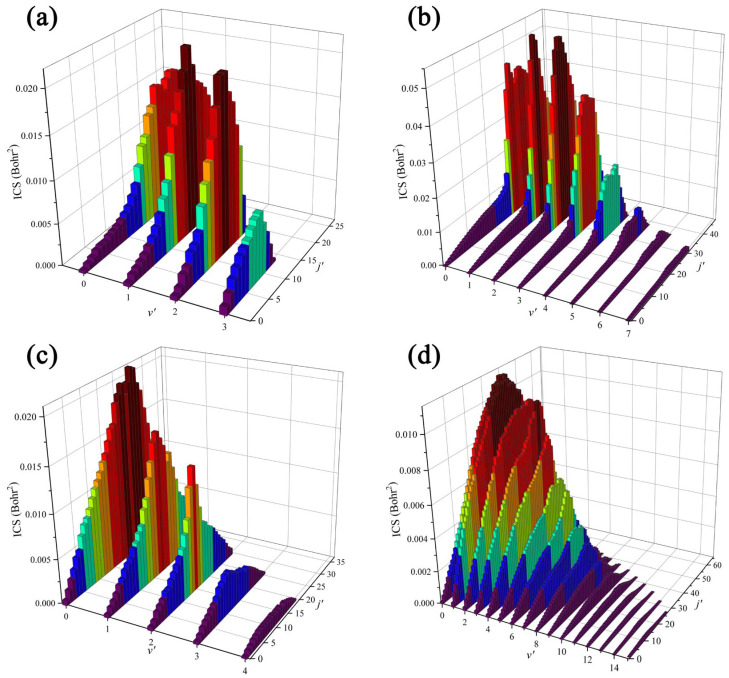
Ro-vibrationally resolved ICSs of the BeH + D channel at (**a**) 2.5 and (**b**) 4.0 eV collision energy and the BeD + H channel at (**c**) 2.5 and (**d**) 4.0 eV for the Be(^1^S) + HD (*v*_0_ = 2, *j*_0_ = 0) → BeH/BeD + H/D reaction.

To further understand the reaction mechanisms and the intramolecular isotope effects of the Be(^1^S) + HD (*v*_0_ = 2, *j*_0_ = 0) reaction at the state-to-state level, the ro-vibrationally resolved ICSs of the two product channels at collision energies of 2.5 and 4.0 eV are also calculated, as shown in Figure 5. The BeD molecule can be excited to a higher rovibrational state than the BeH molecule at the same collision energy due to the smaller vibrational frequency and rotational constant for the former, which correspond to the narrower energy difference between the two adjacent vibrational or rotational energy levels. The maximum availability vibrational and rotational quantum numbers at 2.5 eV for the BeH and BeD molecules are (3, 27) and (4, 34), respectively. For the BeH + D channel, the envelope of rotational states is similar for each vibrational state, and the product is mainly excited to relatively low rotational states. In addition, there exists vibrational population inversion, and this behavior becomes more obvious at 4.0 eV collision energy, indicating that the BeH product is mainly formed by a direct abstraction process, and the dominance of the direct pathway significantly increases as the collision energy increases. On the contrary, the BeD product is distributed at a low vibrational state, and the ICS value gradually decreases as there is an increase in the vibrational quantum number, which is a typical behavior of the statistically dominated process. Compared to the BeH product, the BeD product prefers to populate at higher rotational states, displaying an obvious indirect reaction process. The available rovibrational state of the BeH molecule is much more than the BeD molecule at 4.0 eV collision energy, which is because the BeH + D channel is dominated by the direct pathway, and the contribution of high-order partial waves becomes larger, so a part of the energy is used to overcome the centrifugal barrier, resulting in the BeH molecule not being able to be excited to a higher rovibrational energy level.

**Figure 6:**
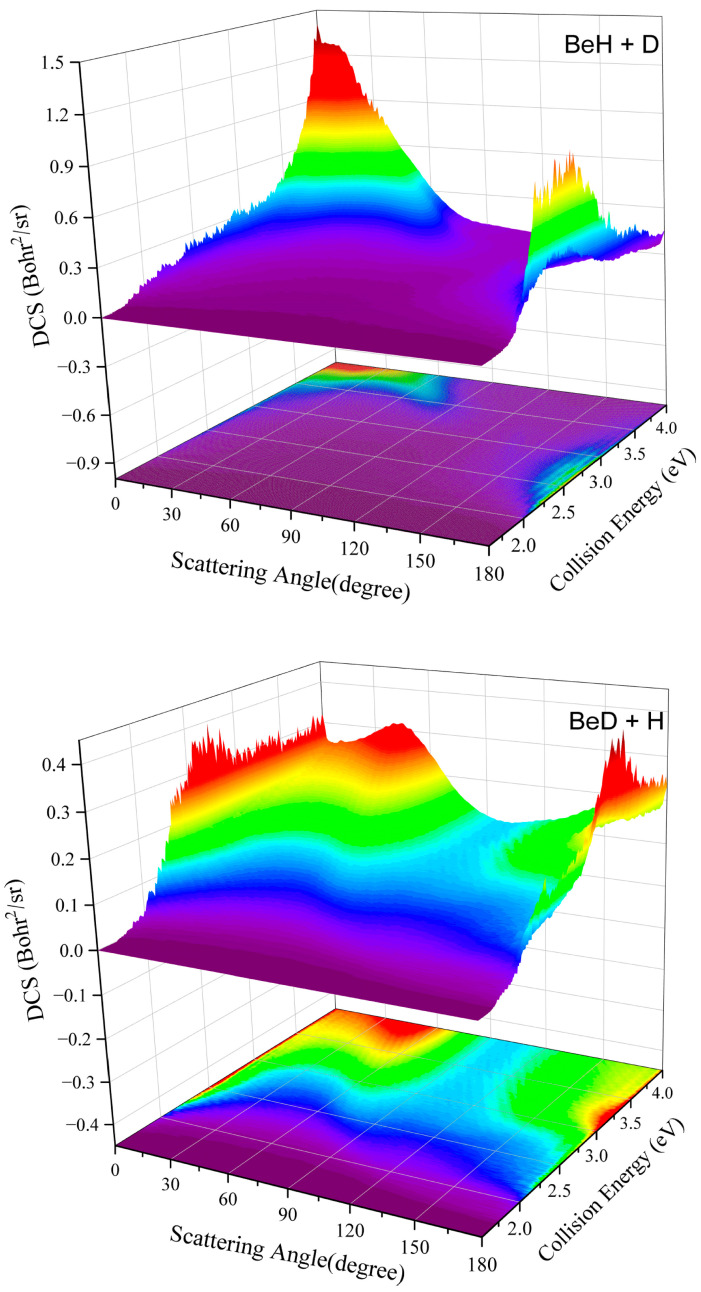
Total DCSs of t the Be(^1^S) + HD (*v*_0_ = 2, *j*_0_ = 0) → BeH/BeD + H/D reaction as a function of collision energy.

The DCSs can more intuitively reveal the microscopic dynamic mechanisms by giving the angle distributions of the product molecules. Figure 6 presents the three-dimensional plots of the total DCSs of the two product channels for the Be(^1^S) + HD (*v*_0_ = 2, *j*_0_ = 0) reaction as a function of collision energy. For both of the product channels, the peaks of the DCSs are distributed at two polar angles and are almost forward–backward symmetric when the collision energy is slightly larger than the threshold, implying the typical statistical nature, which is contributed by the dominance of the deep well on the indirect pathway. As the collision energy increases, the BeD + H channel still keeps the symmetric angle distribution, although a small forward or backward bias exists because of the effect of the centrifugal barrier. However, there is a clear preference for forward or backward scattering on the BeH + D channel, and this behavior is significantly enhanced at high collision energy, displaying obvious nonstatistical features. The remarkable difference in the DCSs between the two product channels suggests that the intramolecular isotope effects are significant in terms of the product angle distributions. The total DCS results further indicate that the BeH + D and BeD + H channels are dominated by the direct abstraction process and complex-forming mechanisms at most of the studied collision energies.

**Figure 7:**
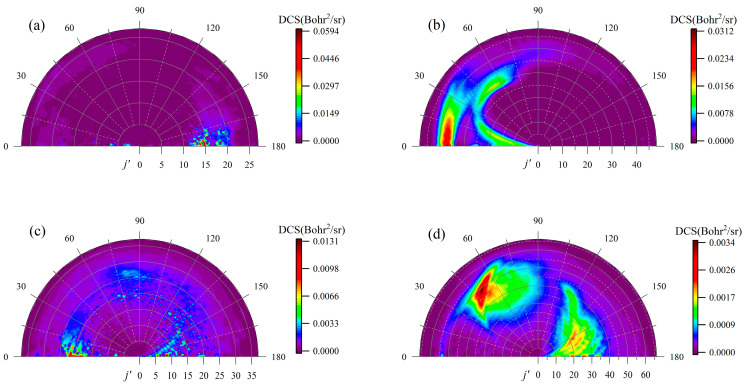
Rotationally resolved DCSs of the BeH + D channel at (**a**) 2.5 and (**b**) 4.0 eV collision energy and the BeD + H channel at (**c**) 2.5 and (**d**) 4.0 eV at *v*′ = 0 for the Be(^1^S) + HD (*v*_0_ = 2, *j*_0_ = 0) → BeH/BeD (*v*′ = 0, *j*′) + H/D reaction.

To present more details about the angle distributions of the title reaction, Figure 7 displays the rotational state-resolved DCSs of the two product channels in the vibrational ground-state for the Be(^1^S) + HD (*v*_0_ = 2, *j*_0_ = 0) reaction at 2.5 eV and 4.0 eV collision energy. The symmetry of the DCSs of the two channels is broken at the rotational state-resolved levels even at low collision energy, which is because the quantum state-resolved dynamics results do not follow the statistical behavior even for long-lived complex-forming reactions [36,37]. The BeH molecules at low rotational states which tend to show forward scattering and backward scattering play dominant roles for the high rotational states at 2.5 eV collision energy. However, the distribution of the rotational state of the BeH product is mainly concentrated in the angle range of 10° to 75° when the collision energy increases to 4.0 eV. For the BeD product, apart from the obvious forward and backward peaks, strong sideways scattering also exists (60–120°), especially at relatively high rotational states.

## 3. Methods

The most reliable strategy for studying reaction dynamics theoretically is to perform quantum mechanics calculations on a high-quality PES [38,39,40]. The quantum TDWP method [41,42,43,44,45] can not only accurately calculate dynamics data but also has a relatively small numerical cost, meaning that it has been widely applied to simple reaction systems [46,47,48,49,50,51,52,53,54,55]. A detailed description of the TDWP method has been presented in the relevant literature, and we only give the essentials and main equations. The Hamiltonian of the Be(^1^S) + HD → BeH/BeD + H/D reaction in the body-fixed (BF) reactant Jacobi coordinates (*r*, *R*, *θ*) can be written as follows:

(1) $$\hat{H}=-\frac{\hbar^{2}}{2\mu_{R}}\frac{\partial^{2}}{\partial R^{2}}-\frac{\hbar^{2}}{2\mu_{r}}\frac{\partial^{2}}{\partial r^{2}}+\frac{{\left(\hat{J}-\hat{j}\right)}^{2}}{2\mu_{R}R^{2}}+\frac{{\hat{j}}^{2}}{2\mu_{r}r^{2}}+\hat{V}(r,R,\theta )$$

where *R* is the distance from the Be atom to the center of the mass of the HD molecule, and *r* is the bond length of the HD molecule. *J* and *j* are the total angle momentum number of the BeHD molecule and the rotational angular momentum number of the HD molecule, respectively. *μ_R_* and *μ_r_* represent the corresponding reduced masses of *R* and *r* coordinates. *V*(*r*, *R*, *θ*) is the Be-HD interaction potential excluding the reference potential energy of the two-body HD molecule. The total wavefunction in the BF representation is expanded to the form of a translational–vibrational–rotational basis, written as follows:

(2) $$\Psi^{JM\varepsilon }(R,r,\theta )=\sum_{nvjK}F_{nvjK}^{JM\varepsilon }D_{MK}^{J\varepsilon }\left(\Omega \right)u_{n}\left(R\right)\phi_{v}(r)y_{jK}\left(\theta \right)$$

where *K* and *M* denote the projection of *J* in the *z* axis of the BF and space-fixed (SF) representations, respectively; $D_{MK}^{J\varepsilon }\left(\Omega \right)$ expresses the parity-adapted normalized Wigner rotation matrix. The initial wave packet in the SF representation contains a Gaussian function $G(R_{\alpha })$, an eigenfunction of HD molecule $\phi_{v_{0}j_{0}}(r_{\alpha })$ with the initial ro-vibrational state (*v*_0_, *j*_0_) and the eigenfunction of total angular momentum $|JMj_{0}l_{0}\varepsilon \rangle$, written as follows:

(3) $$\Psi_{v_{0}j_{0}l_{0}}^{JM\varepsilon }(t=0)=G(R_{\alpha })\phi_{v_{0}j_{0}}(r_{\alpha })|JMj_{0}l_{0}\varepsilon \rangle$$

where $\varepsilon ={\left(-1\right)}^{j_{0}+l_{0}}$ is the parity of the system. The subscript *α* represents the Be(^1^S) + HD channel. The wave packet is evolved by the second-order split operator propagator [56]. During the propagation of the wave packet, *R* and *r* coordinates are multiplied by the damping functions to avoid reflection of the wave function on the boundaries. The final state-to-state S-matrix element is extracted using the reactant-coordinate-based method [57,58]. The reaction probability at the quantum state-resolved level can be calculated by:

(4) $$P_{\upsilon j\leftarrow \upsilon_{0}j_{0}}^{J}=\frac{1}{2j_{0}+1}{\sum_{K,K_{0}}\left|S_{\nu jK\leftarrow \nu_{0}j_{0}K_{0}}^{J}\right|}^{2}$$

The state-to-state integral cross sections (ICSs) and differential cross sections (DCSs) are obtained by using the *S*-matrix, written as

(5) $$\sigma_{\upsilon j\leftarrow \upsilon_{0}j_{0}}=\frac{\pi }{(2j_{0}+1)k_{\upsilon_{0}j_{0}}^{2}}\sum_{K}\sum_{K_{0}}{\sum_{J}(2J+1)\left|S_{\nu jK\leftarrow \nu_{0}j_{0}K_{0}}^{J\epsilon }\right|}^{2}$$

and

(6) $$\frac{d\sigma_{\upsilon j\leftarrow \upsilon_{0}j_{0}}(\vartheta ,E)}{d\Omega }=\frac{1}{\left(2j_{0}+1\right)}{\sum_{K}\sum_{K_{0}}\left|\frac{1}{2ik_{\upsilon_{0}j_{0}}}\sum_{J}(2J+1)d_{KK_{0}}^{J}(\vartheta )S_{\nu jK\leftarrow \nu_{0}j_{0}K_{0}}^{J\epsilon }\right|}^{2}$$

where $k_{v_{0}j_{0}}$ is the moment in the entrance channel, ϑ expresses the scattering angle, and $d_{KK_{0}}^{J}(\vartheta )$ represents the reduced rotation matrix element.

In this work, the initial rovibrational state of the reactant HD molecule is set as *v*_0_ = 2 and *j*_0_ = 0. The main numerical parameters used in the TDWP calculations are listed in Table 1, which are determined by numerous tests. The partial wave is calculated up to *J* = 86, yielding converged ICSs and DCSs for each product channel below 4.0 eV collision energy.

## 4. Conclusions

In the present study, accurate quantum dynamic calculations on the Be(^1^S) + HD (*v*_0_ = 2, *j*_0_ = 0) → BeH/BeD + H/D reaction are carried out at the state-to-state level in the collision energy range up to 4.0 eV using the TDWP method on a globally accurate PES. The detailed dynamics results of the reaction probabilities, opacity functions, and total and state-resolved ICSs and DCSs of the two product channels are displayed to study the reaction mechanisms and intramolecular isotope effects. The title reaction is controlled by two different pathways, namely the indirect pathway, which includes a barrier and a deep well, and a smooth direct abstraction pathway.

There are obvious oscillations in reaction probabilities at small partial waves due to the forming of a collinear HBeD complex. The reaction probabilities of the BeD + H channel decrease with the increasing *J*, but the probabilities have larger values at high partial waves for forming the BeH product. The curves of the opacity functions of the BeH + D channel rise slower with the *J* value and display a more gradual drop after reaching the peak value. The total ICS monotonously increases with the increase in collision energy for both of the channels. The BeH/BeD branching ratio decreases firstly from around one and then rises rapidly, and the preference in generating the BeH molecule is larger than the BeD molecule when the collision energy is above 3.56 eV. The BeH molecule presents vibrational population inversion and is mainly distributed in the low rotational state, whereas the BeD molecule prefers to populate at vibrationally cold and rotationally hot states. The angle distributions are almost forward–backward symmetric when the collision energy is slightly larger than the threshold for both of the product channels. As the collision energy increases, the DCSs of the BeH molecule show an obvious forward or backward bias, while the BeD + H channel remains the symmetric angle distribution. The rotational state-resolved DCSs show nonstatistical characteristics for both of the channels. The dynamics results suggest that the BeH + D channel follows a direct abstraction process at most selected collision energies, while the BeD + H channel is dominated by the complex-forming mechanism.

## Figures and Tables

**Table 1:** Numerical parameters used in the TDWP calculations for Be + HD (*v*_0_ = 2, *j*_0_ = 0) reaction. Atomic units are used if not otherwise stated.

Parameter	Value
*R* *r* Rotational basisInitial wave packet	*R* ∈ [0.1, 15.0], *N_R_* = 199, $N_{R}^{Int}\;$ = 129 *r* ∈ [0.01, 16.0], ν_Int_ = 159, ν_Asy_ = 15 *j*_Int_ = 139, *j*_Asy_ = 39 *R_c_* = 10.0, *δ* = 0.3, *E_c_* = 2.7 eV
Absorbing potential	*R*: *C_a_* = 0. 06, *C_b_* = 0.12, *R_a_* = 11.0, *R_b_* = 15.0
	*r*: *C_a_* = 0.04, *C_b_* = 0.12, *r_a_* = 12.0, *r_b_* = 15.0
Propagation time Matching plane	*T*_tot_ = 40000, Δ*_t_* = 10 *R*_0_′ = 8.0

## Data Availability

The data that support the findings of this study are available from the corresponding author upon reasonable request.
